# Shrunken Hepatic Hemangioma Following Delineated Peritumoral Hyperintensity on Gadoxetic Acid Disodium-enhanced MR Imaging

**DOI:** 10.2463/mrms.ci.2016-0108

**Published:** 2017-02-06

**Authors:** Hiroki Haradome, Takao Okubo, Yusuke Toda, Jun Woo, Tadatoshi Takayama, Osamu Abe

**Affiliations:** 1 Department of Radiology, Nihon University School of Medicine, 30-1 Ohyaguchi, Kami-cho, Itabashi, Tokyo 173-8610, Japan; 2 Department of Digestive Surgery, Nihon University School of Medicine, Tokyo, Japan; 3 Department of Radiology, Graduate School of Medicine, The University of Tokyo, Tokyo, Japan

**Keywords:** hemangioma, liver, peritumoral hyperplasia, magnetic resonance imaging, gadoxetic acid disodium

## Introduction

Most hepatic hemangiomas remain stable on follow-up imaging. Rarely, they might be accompanied by hemorrhage, infarction, thrombosis, calcification, and sclerosis. Among these secondary histological alterations, a sclerosed/sclerosing hepatic hemangioma, characterized by massive fibrosis, hyalinization, and nearly complete obliteration of the vascular cavities, is a known rare variant that spontaneously shrinks.^1^

Recently, peritumoral hyperintensity on the hepatocyte phase of gadoxetic acid disodium (EOB)-enhanced magnetic resonance (MR) imaging was reported to correspond to peritumoral hyperplasia with high glutamine synthetase and the expression of the organic anion transporter polypeptide 1B3; furthermore, peritumoral hyperintensity is occasionally observed with malignant hepatic tumors.^2^ However, it has not been described previously with benign hepatic tumors.

**Fig 1.**
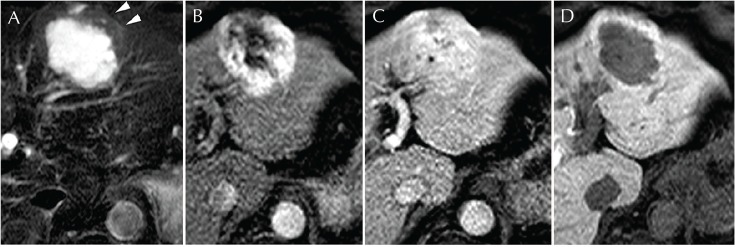
(**A**) Fat-saturated T_2_-weighted magnetic resonance (MR) image shows a nodule with strong hyperintensity and surrounding faint hyperintense area (arrowheads). (**B**, **C**) The arterial and portal phases of a gadoxetic acid disodium-enhanced MR images reveal a nodule with progressive, peripheral enhancement, and it appears hypointense with circumferential peritumoral hyperintense on (**D**) the hepatocyte phase.

**Fig 2.**
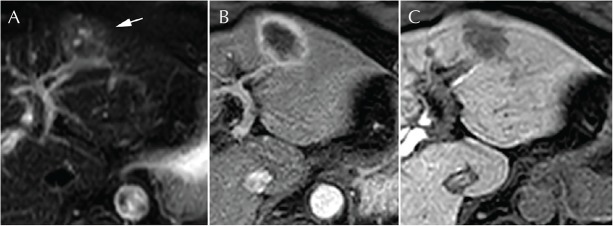
(**A**) The fat-saturated T_2_-weighted MR image reveals the shrunken nodule showing a dark signal (arrow) at follow-up examination after 21 months. (**B**) In addition, the arterial phase of the EOB-enhanced MR image reveals ring-enhancement, which is suspicious for its sclerosed change. (**C**) The hepatocyte phase of the EOB-enhanced MR demonstrates the hypointense-appearing nodule, but peritumoral hyperintensity is less prominent at this time.

A 75-year-old asymptomatic man periodically attended our hospital for the follow-up of a hepatic hemangioma, which was incidentally discovered by abdominal ultrasound examination in December 2009. Hepatitis-related indexes and tumor markers were negative. At the time of follow-up MR imaging in November 2014, the nodule with a diameter of 52 mm showed strong hyperintensity and the faint hyperintense area around it on fat-saturated (FS) T_2_-weighted image (Fig. 1A). EOB-enhanced MR imaging revealed a progressive, peripheral enhancement from arterial to portal phases (Fig. 1B, C), which was consistent with a hemangioma; additionally, the nodule was hypointense with circumferential peritumoral hyperintensity on the hepatocyte phase (Fig. 1D). On follow-up MR imaging after 21 months, the nodule had shrunk significantly to 27 mm in diameter. The signal on the fat-saturated T_2_-weighted image (Fig. 2A) was mostly dark, showing ring-enhancement from arterial to late phases (Fig. 2B) and hypointensity on the hepatocyte phase, but peritumoral hyperintensity was less prominent at this time (Fig. 2C).

In the present case, an intriguing size change was observed during the follow-up imaging examination. The hemangioma decreased relatively rapidly to about half of the previous size after delineating the circumferential peritumoral hyperintensity on the hepatocyte phase of EOB-enhanced MR imaging. In addition to the reduced size, the imaging features of a typical hemangioma changed into them, which were suspicious for a sclerosed hemangioma that is signaled on a T_2_-weighted image by becoming dark, probably reflecting fibrosis and it showed a ring-enhancement from arterial to late phases.^1^ Recently, it has been assumed that peritumoral hyperintensity on the hepatocyte phase of an EOB-enhanced MR image corresponds to peritumoral hyperplasia on pathological examination.^2^ Peritumoral hyperplasia has been reportedly observed in various malignant hepatic tumors including hepatocellular carcinomas (usual or fibrolamellar type), hepatoblastomas, or hepatic metastases from neuroendocrine tumors, gastrointestinal stromal tumors, and colonic adenocarcinomas.^3^ The pathogenesis of the condition is thought to include the following: 1) increased arterial blood flow due to portal vein invasion; and 2) specific tumor-secreted hormones or growth factors, which can induce hepatocyte proliferation.^3^ In the present case, the circumferential peritumoral hyperintensity was observed around a hemangioma prior to the reduced size, presumably due to sclerotic changes. Furthermore, it is speculated that regional blood flow alterations induced by thrombosed small vascular cavities may account for the peritumoral hyperintensity (hyperplasia). More interestingly, the faint hyperintense area around the hemangioma was observed on FS-T_2_-weighted image (Fig. 1A), which mostly conformed peripheral arterial enhanced portion (Fig. 1B) and circumferential peritumoral hyperintensity of the hepatocyte phase (Fig. 1D) on EOB-enhanced MR images. On an apparent diffusion coefficient (ADC) map, an area corresponding to the faint hyperintese signal on FS-T_2_-weighted image showed slightly higher ADC value (1.5 × 10^−3^ mm^2^/s) compared to that of the normal liver parenchyma (1.3 × 10^−3^ mm^2^/s; the hemangioma, 2.4 × 10^−3^ mm^2^/s) therefore it was presumed that some edematous change and increased vascular bed reflecting hyperperfusion state might contribute the faint peritumoral hyperintense on FS-T_2_-weighted image and this feature became less obvious when the hamangioma shrank (Fig. 2A).

In summary, peritumoral hyperintensity on the hepatocyte phase of EOB-enhanced MR imaging is observed even in hemangiomas. The peritumoral hyperintensity may predict some regional blood flow alteration reflecting secondary histological changes.
